# Thermodynamic Properties of a Regular Black Hole in Gravity Coupling to Nonlinear Electrodynamics

**DOI:** 10.3390/e20030192

**Published:** 2018-03-13

**Authors:** Yi-Huan Wei

**Affiliations:** Department of Physics, Bohai University, Jinzhou 121000, China; yihuanwei@126.com

**Keywords:** thermodynamic properties, ruppeiner thermodynamic geometry, NED black hole, 98.80.-k, 04.62.+v

## Abstract

We first calculate the heat capacities of the nonlinear electrodynamics (NED) black hole for fixed mass and electric charge, and the electric capacitances for fixed mass and entropy. Then, we study the properties of the Ruppeiner thermodynamic geometry of the NED black hole. Lastly, some discussions on the thermal stability of the NED black hole and the implication to the flatness of its Ruppeiner thermodynamic geometry are given.

## 1. Introduction

The thermodynamics of black holes are established on the basis of the definitions of the entropy and temperature of black hole [1,2,3,4]. The temperature of the horizon of a black hole is proportional to its surface gravity. In Einstein gravity, the entropy of the horizon of a black hole is proportional to its horizon area (the entropy area law of a black hole). Using the brick-wall method [5], one can check the entropy area law of a black hole. However, the result from the brick-wall model depends on the choice for the membrane near the horizon. Whether the entropy of a black hole satisfies the entropy area law will depend on the adopted gravity theory.

For the thermodynamic system described by the *N* thermodynamic variables including the entropy, the internal energy, and so on, one can construct the corresponding thermodynamic geometry associated with the entropy, the internal energy and other thermodynamic variable [6,7,8,9]. The Ruppeiner geometry can reflect the fluctuation property of an equilibrium thermodynamic system. The curvature scalar of the Ruppeiner geometry is related to the correlation volume of a thermodynamic system, and its divergent point is the critical point of the thermodynamic phase transition. The properties of the thermodynamic geometries of the black hole have been widely studied [10,11,12,13,14,15,16,17,18,19,20,21,22,23,24,25,26,27,28,29,30,31,32,33,34,35,36,37,38]. It has been found that the Ruppeiner thermodynamic geometry of the Reissner–Nordström (RN) black hole is flat [37]. On the other hand, some regular black hole solutions have been found in the gravity theory coupling to nonlinear electrodynamics (NED) [39,40,41,42]. The regular NED black hole with exponential mass function approaches to the RN black hole asymptotically [40]. The entropy of black hole depends on the action of gravity theory [43,44,45,46,47,48]. In the NED gravity theory, the entropy of a black hole is still the Bekenstein entropy [1,2,49]. So, it is expected that, like the Ruppeiner thermodynamic geometry of the RN black hole, the Ruppeiner thermodynamic geometry of the regular NED black hole with exponential mass function will be flat.

In this paper, we study the thermodynamic properties and the properties of thermodynamic geometry of the regular NED black hole. In Section 2, we calculate the heat capacity and the electric capacitance of the NED black hole. In Section 3, we study the properties of the Ruppeiner thermodynamic geometry of NED black hole. Lastly, we discuss some problems on the thermodynamic properties of the NED black hole.

## 2. Thermodynamic Properties of NED Black Hole

The action of Einstein gravity coupling to the nonlinear electrodynamics is [50]

(1) $$\begin{array}{c} S=\frac{1}{16\pi }\int d^{4}x\sqrt{-g}\left(R+4L\left(F\right)\right), \end{array}$$

where *g* and *R* are the metric determinant and Ricci curvature scalar associated with metric $g_{\mu \nu }$, L=L(F) is the Lagrangian of the NED source with $F=\frac{1}{4}F_{\mu \nu }F^{\mu \nu }$. By defining $P=\frac{1}{4}P_{\mu \nu }P^{\mu \nu }$ with $P_{\mu \nu }=L_{F}F_{\mu \nu }$, then one has the Legendre transformation

(2) $$\begin{array}{c} L=2PH_{P}-H,\;H=2FL_{F}-L, \end{array}$$

where H=H(P) is a function of *P*, $L_{F}=\frac{dL}{dF}$ and $H_{P}=\frac{dH}{dP}$. In the dual H representation, the energy-momentum tensor is given as

(3) $$\begin{array}{c} T_{\mu \nu }=\frac{1}{4\pi }H_{P}P_{\mu \lambda }P_{\nu }^{\lambda }-\frac{1}{4\pi }g_{\mu \nu }\left(2PH_{P}-H\right). \end{array}$$

Recently, some regular solutions in Einstein gravity coupling to the nonlinear electrodynamics have been obtained [51,52]. For the static, spherically symmetric metric

(4) $$\begin{array}{c} ds^{2}=-fdt^{2}+f^{-1}dr^{2}+r^{2}\left(d\theta^{2}+sin^{2}\varphi d\varphi^{2}\right), \end{array}$$

where $f=1-\frac{2m(r)}{r}$ with m(r) the mass function, the Einstein field equation yields [40]

(5) $$\begin{array}{c} m\left(r\right)=\int -r^{2}Hdr. \end{array}$$

**Figure 1:**
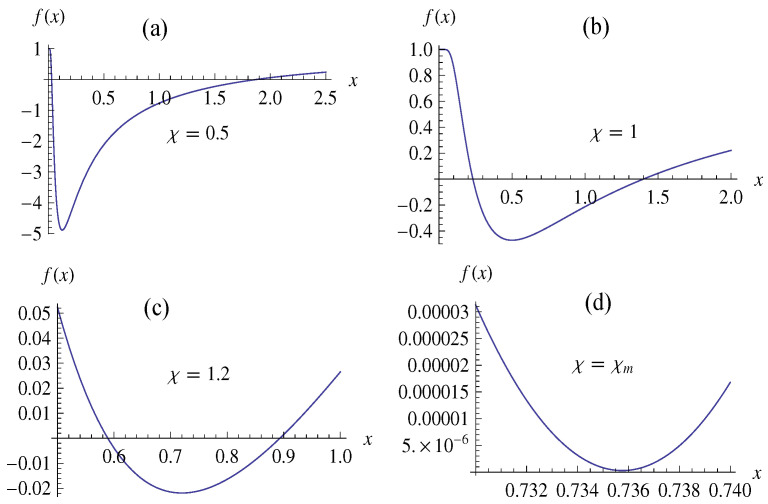
The metric function f(x) with $x=\frac{r}{M}$ for the four cases: (**a**) χ=0.5; (**b**) χ=1.0, (**c**) χ=1.2, (**d**) χ=1.2130615.

For

(6) $$\begin{array}{c} H=Pe^{-U},\;U=\frac{q}{2M}\sqrt{-2q^{2}P}, \end{array}$$

the mass function takes the exponential form

(7) $$\begin{array}{c} m\left(r\right)=Me^{-\frac{q^{2}}{2Mr}}, \end{array}$$

where *M* and *q* are the mass and electric charge of the NED spacetime. The NED black hole possesses the two horizons with the outer (inner) horizon radius $r_{+}$ ($r_{-}$). For the ratio $\chi =q/M=\chi_{m}$ with $\chi_{m}=2/\sqrt{e}$, the two horizons of NED black hole coincide, as shown in Figure 1.

In the following, we will discuss the thermal and electric properties of the NED black hole. From

(8) $$\begin{array}{c} T_{+}=\frac{1}{4\pi }\frac{df}{dr}{|}_{r=r_{+}}, \end{array}$$

the outer horizon temperature of the NED black hole $T_{NED+}$ is obtained as

(9) $$\begin{array}{c} T_{NED+}=\frac{2Mr_{+}-q^{2}}{8\pi Mr_{+}^{2}}. \end{array}$$

From $f(r_{+})=0$, one gets the mass of NED spacetime. The mass M includes the mass of NED black hole (the mass enclosed by the outer horizon of NED black hole) and the mass outside the outer horizon of NED black hole.

(10) $$\begin{array}{c} M=\frac{1}{2}r_{+}e^{\frac{q^{2}}{2Mr_{+}}}, \end{array}$$

which approaches $\frac{1}{2}r_{+}$ as χ→0. The Misner-Sharp energy enclosed by the sphere surface of radius *r* in the spherically symmetric spacetime takes [53]

(11) $$\begin{array}{c} E_{NED}=\frac{1}{2}r\left(1-f\right). \end{array}$$

Putting $f(r_{+})=0$ in Equation (11) gives

(12) $$\begin{array}{c} E_{NED+}=\frac{1}{2}r_{+}, \end{array}$$

where $E_{NED+}$ denotes the energy inside the outer horizon of the NED black hole, which is less than the mass of NED spacetime.

In the process that the outer horizon radius of the NED black hole increases $dr_{+}$, the energy $E_{NED+}$ correspondingly increases $dE_{NED+}=\frac{1}{2}dr_{+}$. In this process, the entropy of the outer horizon increases $dS_{NED+}=2\pi r_{+}dr_{+}$ and the heat energy flowing into the black hole through the outer horizon is $dQ=T_{NED+}dS_{NED+}$. According to Ref. [54], the volume enclosed by the outer horizon of the NED black hole is given as $V_{NED+}=\frac{4}{3}\pi r_{+}^{3}$. The work done by the outer horizon of the NED black hole is $dA=p_{NED+}dV_{NED+}$, where $p_{NED+}=-\frac{q^{2}}{16\pi Mr_{+}^{3}}$ is the radial pressure on the outer horizon. As a result, the first law of thermodynamics for the NED black hole is expressed as

(13) $$\begin{array}{c} dE_{NED+}=dQ-dA=T_{NED+}dS_{NED+}-p_{NED+}dV_{NED+}. \end{array}$$

The thermal stability of the NED black hole may be checked by determining the sign of its heat capacity [55]. The heat capacity of the NED black hole associated with the outer horizon is defined as [56]

(14) $$\begin{array}{c} C_{+X}=T_{+}{\left(\frac{\partial S_{+}}{\partial T_{+}}\right)}_{X}=2\pi r_{+}T_{+}{\left(\frac{\partial r_{+}}{\partial T_{+}}\right)}_{X}, \end{array}$$

where the subscript *X* denotes the thermodynamic process with thermodynamic quantity *X* fixed. The heat capacity defined by Equation (14) differs from the one given by Equation (58) in Ref. [57]. For the latter definition, the heat capacity for a fixed *M* is zero.

According to Equation (14), the heat capacity of the NED black for a fixed *M* is obtained as

(15) $$\begin{array}{c} C_{+M}=\frac{2\pi r_{+}^{2}\left(2Mr_{+}-q^{2}\right)}{q^{2}}. \end{array}$$

For a fixed *q*, the heat capacity is given as

(16) $$\begin{array}{c} C_{+q}=\frac{2\pi r_{+}^{2}\left(4M^{2}r_{+}^{2}-q^{4}\right)}{4M^{2}r_{+}^{2}-4Mq^{2}r_{+}-q^{4}}. \end{array}$$

Letting $y_{+}=\ln\left[\frac{1}{2}x_{+}\right]$ with $x_{+}=\frac{r_{+}}{M}$, then there is $-1\leq y_{+}\leq 0$ ($0.736<x_{+}\leq 2$). Substituting $q^{2}=-2Mr_{+}y_{+}$ into Equations (15) and (16) yields

(17) $$\begin{array}{c} C_{+M}=-2\pi r_{+}^{2}\left[1+y_{+}^{-1}\right], \end{array}$$

(18) $$\begin{array}{c} C_{+q}=\frac{2\pi r_{+}^{2}\left(1-y_{+}^{2}\right)}{y_{+}^{2}-2y_{+}-1}. \end{array}$$

Clearly, $C_{+M}$ is non-negative and thus the NED black hole is thermally stable in the process with *M* fixed. At $y_{+}=y_{+}^{\left(1\right)}≃-0.414$, $C_{+q}$ is divergent, and for $y_{+}<y_{+}^{\left(1\right)}$ it is positive.

From the formula for the electrostatic field $E=-\frac{r^{3}}{2q}\frac{d}{dr}\left(\frac{1}{r^{2}}\frac{d\sigma }{dr}\right)$ [40], the electric field of NED black hole is obtained as

(19) $$\begin{array}{c} E=\frac{8Mqr-q^{3}}{8Mr^{3}}e^{-\frac{q^{2}}{2Mr}}. \end{array}$$

Integrating the electric field *E* gives the electric potential of the NED black hole as [49]

(20) $$\begin{array}{c} \Phi \left(r\right)=-\int Edr=\frac{q^{2}-6Mr}{4qr}e^{-\frac{q^{2}}{2Mr}}+\tilde{C}, \end{array}$$

with $\tilde{C}$ an integration constant. Taking Φ(0)=0, then $\tilde{C}=0$ and $\Phi \left(\infty \right)=-\frac{3M}{2q}$. Under this boundary condition, the electric potential on the outer horizon of the NED black hole is given as

(21) $$\begin{array}{c} \Phi_{+}=\Phi \left(r_{+}\right)=\frac{q^{2}-6Mr_{+}}{8Mq}. \end{array}$$

For the extremal black hole, the electric potential on the horizon of the NED black hole reduces to $\Phi_{ext}=-\frac{q}{4M}=-\frac{1}{2\sqrt{e}}$.

The electric capacitance of the NED black hole is defined as

(22) $$\begin{array}{c} K_{+X}=\beta_{+}{\left(\frac{\partial q}{\partial \phi_{+}}\right)}_{X}, \end{array}$$

where $\beta_{+}=T_{+}^{-1}$ is the inverse temperature of the outer horizon of the NED black hole and $\phi_{+}$ is defined as

(23) $$\begin{array}{c} \phi_{+}=\beta_{+}\Phi_{+}=\frac{\pi r_{+}^{2}\left(q^{2}-6Mr_{+}\right)}{q^{3}-2Mqr_{+}}. \end{array}$$

For a fixed *M*, the electric capacitance of the NED black hole is obtained as

(24) $$\begin{array}{c} K_{+M}=\frac{8M{\left(q^{3}-2Mqr_{+}\right)}^{2}}{3q^{6}-22Mq^{4}r_{+}+4M^{2}q^{2}r_{+}^{2}+24M^{3}r_{+}^{3}}, \end{array}$$

or

(25) $$\begin{array}{c} K_{+M}=\frac{8My_{+}{\left(1+y_{+}\right)}^{2}}{3y_{+}^{3}+11y_{+}^{2}+y_{+}-3}. \end{array}$$

As $y_{+}\to -1$, there is $K_{+M}\to 0^{+}$. At $y_{+}=y_{+}^{\left(2\right)}≃-0.632$, $K_{+M}$ is divergent and it is positive for $y_{+}>y_{+}^{\left(2\right)}$. For a fixed $S_{+}$, the electric capacitance of the NED black hole is

(26) $$\begin{array}{c} K_{+S}=\frac{8Mq^{2}\left(4M^{2}r_{+}^{2}-q^{4}\right)}{q^{6}-6Mq^{4}r_{+}-20M^{2}q^{2}r_{+}^{2}+24M^{3}r_{+}^{3}}, \end{array}$$

or

(27) $$\begin{array}{c} K_{+S}=\frac{8My_{+}\left(1-y_{+}^{2}\right)}{y_{+}^{3}+3y_{+}^{2}-5y_{+}-3}. \end{array}$$

As $y_{+}\to -1$, $K_{+S}\to 0^{+}$. $K_{+S}$ is divergent at $y_{+}=y_{+}^{\left(3\right)}≃-0.483$, and is positive for $y_{+}>y_{+}^{\left(3\right)}$. Equations (25) and (27) show that the NED black hole with a small charge/mass ratio is electrostatically stable in the process with *M* fixed and the one with $S_{+}$ fixed.

The NED black hole possesses the two horizons for $\chi <\chi_{m}$. The observer outside the outer horizon may detect the thermal properties of the outer horizon. We live in the spacetime with a cosmological horizon lying in the outside of us. Similarly, one can imagine that there are some observers inside the inner horizon of the NED black hole. For such a kind of observers, the inner horizon will be of equal importance as the outer horizon for us. In order to get a complete understanding for the thermal properties of the black hole with two horizons, we also need to study the thermal properties of its inner horizon.

The entropy and temperature of the inner horizon of the NED black hole are $S_{NED-}=\pi r_{-}^{2}$ and $T_{NED-}=\frac{2Mr_{-}-q^{2}}{8\pi Mr_{-}^{2}}$, respectively. From $p_{NED-}=-\rho_{NED}\left(r_{-}\right)$, the radial pressure on the inner horizon of the NED black hole is given as $p_{NED-}=-\frac{q^{2}}{16\pi Mr_{-}^{3}}$. Similar to the case of the outer horizon, the first law of thermodynamics for the inner horizon of NED black hole is

(28) $$\begin{array}{c} dE_{NED-}=T_{NED-}dS_{NED-}-p_{NED-}dV_{-}, \end{array}$$

where $E_{NED-}=\frac{1}{2}r_{-}$ is the energy inside the inner horizon of the NED black hole and $V_{-}=\frac{4}{3}\pi r_{-}^{3}$ is the volume enclosed by the inner horizon of the NED black hole [54].

In terms of $y_{-}=\ln\frac{r_{-}}{2M}$ with $y_{-}<-1$, $T_{NED-}$ is expressed as

(29) $$\begin{array}{c} T_{NED-}=\frac{1+y_{-}}{4\pi r_{-}}, \end{array}$$

which is a negative temperature. The two heat capacities of the NED black hole associated with the inner horizon are

(30) $$\begin{array}{c} C_{-M}=-2\pi r_{-}^{2}\left[1+y_{-}^{-1}\right], \end{array}$$

(31) $$\begin{array}{c} C_{-q}=\frac{2\pi r_{-}^{2}\left(1-y_{-}^{2}\right)}{y_{-}^{2}-2y_{-}-1}. \end{array}$$

Clearly, both $C_{-M}$ and $C_{-q}$ are negative definite. This implies that the inner horizon of the NED black hole is thermally unstable.

Putting $r=r_{-}$ in Equation (20) with $\tilde{C}=0$ gives the electric potential on the inner horizon of the NED black hole as

(32) $$\begin{array}{c} \Phi_{-}=\frac{q^{2}-6Mr_{-}}{8Mq}. \end{array}$$

The two electric capacities of the NED black hole associated with the inner horizon are

(33) $$\begin{array}{c} K_{-M}=\frac{8My_{-}{\left(1+y_{-}\right)}^{2}}{3y_{-}^{3}+11y_{-}^{2}+y_{-}-3}, \end{array}$$

(34) $$\begin{array}{c} K_{-S}=\frac{8My_{-}\left(1-y_{-}^{2}\right)}{y_{-}^{3}+3y_{-}^{2}-5y_{-}-3}. \end{array}$$

At $y_{-}=y_{-}^{\left(2\right)}≃-3.49$, $K_{-M}$ is divergent, and it is positive for $y_{<}y_{-}^{\left(2\right)}$. At $y_{-}=y_{-}^{\left(3\right)}≃-4.05$, $K_{-S}$ is divergent, and it is positive for $y_{>}y_{-}^{\left(3\right)}$.

## 3. Thermodynamic Geometry of NED Black Hole

Some thermodynamic properties of the thermodynamic system may be studied by the thermodynamic geometry method [6,7,8]. The Ruppeiner thermodynamic metric takes the following form [9]

(35) $$\begin{array}{c} dS_{R}^{2}=-\frac{\partial^{2}S}{\partial X^{i}\partial X^{j}}dX^{i}dX^{j}=g_{ij}^{R}dX^{i}dX^{j},\;i,j=1,2, \end{array}$$

where *S* is the entropy of the thermodynamic system, and $X_{i}$ (i=1,2,…) denote the other thermodynamic quantities.

For the black hole with two horizons described by the two thermodynamic quantities, the Ruppeiner metric associated with the entropy of the outer horizon takes the form

(36) $$\begin{array}{c} g_{+}^{R}=\left(g_{+ij}^{R}\right)=-\left(\begin{array}{cc} \frac{\partial^{2}S_{+}}{\partial X_{1}\partial X_{1}} & \frac{\partial^{2}S_{+}}{\partial X_{1}\partial X_{2}}\\ \frac{\partial^{2}S_{+}}{\partial X_{2}\partial X_{1}} & \frac{\partial^{2}S_{+}}{\partial X_{2}\partial X_{2}} \end{array}\right), \end{array}$$

with $\left(X_{1},X_{2}\right)=\left(M,q\right)$ and i(j)=1,2. According to Equation (36), the Ruppeiner metric matrix of the NED black hole is obtained as

(37) $$\begin{array}{c} g_{+}^{R}=\left(\begin{array}{cc} \frac{2\pi r_{+}^{2}\left(3q^{6}+2Mq^{4}r_{+}+4M^{2}q^{2}r_{+}^{2}-8M^{3}r_{+}^{3}\right)}{M^{2}{\left(-q^{2}+2Mr_{+}\right)}^{3}} & -\frac{8\pi q^{5}r_{+}^{2}}{M{\left(-q^{2}+2Mr_{+}\right)}^{3}}\\ -\frac{8\pi q^{5}r_{+}^{2}}{M{\left(-q^{2}+2Mr_{+}\right)}^{3}} & -\frac{4\pi r_{+}^{2}\left(3q^{4}-4Mq^{2}r_{+}+4M^{2}r_{+}^{2}\right)}{{\left(q^{2}-2Mr_{+}\right)}^{3}} \end{array}\right), \end{array}$$

with the determinant $detg_{+}^{R}=-\frac{8\pi^{2}r_{+}^{4}\left(q^{4}+4M^{2}r_{+}^{2}\right)}{M^{2}{\left(-q^{2}+2Mr_{+}\right)}^{3}}$.

The components of Christoffel connection associated with the Ruppeiner metric (36) read

(38) $$\begin{array}{c} \Gamma_{11}^{1}=\frac{FA_{,1}-BA_{,2}}{2(AF-B^{2})},\;\Gamma_{12}^{1}=\frac{FA_{,2}-BF_{,1}}{2(AF-B^{2})},\;\Gamma_{22}^{1}=\frac{FB_{,2}-BF_{,2}}{2(AF-B^{2})}, \end{array}$$

(39) $$\begin{array}{c} \Gamma_{11}^{2}=\frac{AB_{,1}-BA_{,1}}{2(AF-B^{2})},\;\Gamma_{12}^{2}=\frac{AF_{,1}-BA_{,2}}{2(AF-B^{2})},\;\Gamma_{22}^{2}=\frac{AF_{,2}-BF_{,1}}{2(AF-B^{2})}, \end{array}$$

where $A=g_{+11}^{R}$, $B=g_{+12}^{R}$ and $F=g_{+22}^{R}$. For the Ruppeiner metric (37), the components of Christoffel connection are

(40) $$\begin{array}{c} \Gamma_{11}^{1}=\frac{2q^{6}\left(3q^{4}-4Mq^{2}r_{+}+20M^{2}r_{+}^{2}\right)}{M{\left(-q^{2}+2Mr_{+}\right)}^{3}\left(q^{4}+4M^{2}r_{+}^{2}\right)}, \end{array}$$

(41) $$\begin{array}{c} \Gamma_{12}^{1}=\frac{2q^{5}\left(3q^{4}-4Mq^{2}r_{+}+20M^{2}r_{+}^{2}\right)}{{\left(q^{2}-2Mr_{+}\right)}^{3}\left(q^{4}+4M^{2}r_{+}^{2}\right)}, \end{array}$$

(42) $$\begin{array}{c} \Gamma_{22}^{1}=-\frac{2Mq^{4}\left(3q^{4}-4Mq^{2}r_{+}+20M^{2}r_{+}^{2}\right)}{{\left(q^{2}-2Mr_{+}\right)}^{3}\left(q^{4}+4M^{2}r_{+}^{2}\right)}, \end{array}$$

(43) $$\begin{array}{c} \Gamma_{11}^{2}=\frac{q^{5}\left(q^{2}+2Mr_{+}\right)\left(3q^{4}-4Mq^{2}r_{+}+20M^{2}r_{+}^{2}\right)}{M^{2}{\left(-q^{2}+2Mr_{+}\right)}^{3}\left(q^{4}+4M^{2}r_{+}^{2}\right)}, \end{array}$$

(44) $$\begin{array}{c} \Gamma_{12}^{2}=-\frac{q^{4}\left(3q^{6}+2Mq^{4}r_{+}+12M^{2}q^{2}r_{+}^{2}+40M^{3}r_{+}^{3}\right)}{M{\left(-q^{2}+2Mr_{+}\right)}^{3}\left(q^{4}+4M^{2}r_{+}^{2}\right)}, \end{array}$$

(45) $$\begin{array}{c} \Gamma_{22}^{2}=-\frac{q^{3}\left(3q^{6}+2Mq^{4}r_{+}+12M^{2}q^{2}r_{+}^{2}+40M^{3}r_{+}^{3}\right)}{{\left(q^{2}-2Mr_{+}\right)}^{3}\left(q^{4}+4M^{2}r_{+}^{2}\right)}. \end{array}$$

The Ricci tensor of the Ruppeiner thermodynamic geometry associated with the Ruppeiner metric (36) are

(46) $$\begin{array}{c} R_{11}^{R}=AR,\;R_{12}^{R}=R_{21}^{R}=BR,\;R_{22}^{R}=FR, \end{array}$$

where

(47) $$\begin{array}{c} R=\frac{A\left[F_{,2}B_{,1}-{\left(F_{,1}\right)}^{2}\right]+B\left(A_{,2}B_{,2}-F_{,2}A_{,1}\right)+F\left[A_{,1}F_{,1}-{\left(A_{,2}\right)}^{2}\right]}{4{\left(AF-B^{2}\right)}^{2}}. \end{array}$$

For the Ruppeiner metric (37), all the components of the Ricci tensor are zero and thus the Ricci scalar of the Ruppeiner thermodynamic geometry is zero.

Similarly, one can construct the Ruppeiner thermodynamic geometry metric associated with the inner horizon of the NED black hole as $g_{-ij}^{R}=\frac{\partial^{2}S_{-}}{\partial X_{i}\partial X_{j}}$. The corresponding Christoffel connection components may be obtained from Equations (40)–(45) under the substitution $r_{+}\to r_{-}$. So, the Ricci tensor of the Ruppeiner thermodynamic geometry associated with the inner horizon of the NED black hole is also zero.

## 4. Discussions

The thermodynamic properties and the Ruppeiner thermodynamic geometry of the NED black hole have been studied. The thermodynamic systems related to the outer horizon and the inner horizon of the NED black hole satisfy the first law of thermodynamics with the internal energy $E_{NED\pm }=\frac{1}{2}r_{\pm }$. It should be mentioned that the NED black hole releases heat energy when the inner horizon entropy increases, since the inner horizon temperature is negative. Equations (17) and (18) shows that the outer horizon of the NED black hole with a big χ is thermally stable. Equations (30) and (31) means that the inner horizon of the NED black hole is always thermally unstable, since there are $C_{-M}<0$ and $C_{-q}<0$ for an arbitrary value of $\chi <\chi_{m}$. For a black hole with two horizons, there are two effective thermodynamic systems. One system associated with outer horizon may be described by the thermodynamic quantities $S_{NED+}$, *M* and *q*. The other associated with inner horizon may be described by $S_{NED-}$, *M* and *q*. Both of the Ruppeiner thermodynamic geometries associated with the outer and inner horizons of the NED black hole are flat. This reflects that there is no interaction between the particles in the two thermodynamic systems [58]. As a result, there exists no thermodynamic phase transition of the NED black hole.
